# Levodopa-Responsive Parkinsonian Syndrome Secondary to a Compressive Craniopharyngioma: A Case Report

**DOI:** 10.7759/cureus.35621

**Published:** 2023-02-28

**Authors:** Wilson Rodriguez, Margarita Fedorova, Pratap Chand

**Affiliations:** 1 Neurology, Saint Louis University School of Medicine, Saint Louis, USA

**Keywords:** drug-induced parkinsonism, parkinsonian presentation, dopamine replacement therapy, dat scan, primary brain tumor, levodopa-carbidopa, craniopharyngioma, secondary parkinsonism

## Abstract

Parkinsonism is a rare manifestation of brain tumors that has most commonly been reported in association with gliomas and meningiomas. In this paper, we describe a unique case of secondary Parkinsonism that was precipitated by a craniopharyngioma. A 42-year-old female presented with resting tremors, rigidity, and bradykinesia. Her past medical history was significant for a craniopharyngioma resection four months prior. The postoperative course was complicated by severe delirium, panhypopituitarism, and diabetes insipidus. Notably, she was taking haloperidol and aripiprazole daily for four months to manage her delirium and psychotic episodes. Her preoperative brain MRI showed a compressive effect of the craniopharyngioma on the midbrain and nigrostriatum. Drug-induced Parkinsonism was initially suspected given extended treatment with antipsychotics. Haloperidol and aripiprazole were stopped, and benztropine was started with no improvement. Consequently, the patient was treated with carbidopa/levodopa with symptomatic improvement. A dopamine transporter (DaT) scan was done after starting carbidopa/levodopa and showed asymmetric decreased uptake in dopamine transporter in the striatum. Only one other case of Parkinsonism following craniopharyngioma resection was found in the literature review. Unlike our example, the symptoms resolved following surgical intervention and did not require a long-term treatment with carbidopa/levodopa. The purpose of our case report is to highlight brain tumors as a potential cause of secondary Parkinsonism in younger patients for an early surgical intervention can be curative.

## Introduction

Parkinsonism is a motor syndrome manifested by rigidity, tremors, and bradykinesia [1]. In over 80% of patients, it is caused by Parkinson’s disease (PD) but can be due to other etiologies, such as medications or toxins. Parkinsonism due to PD is defined as primary Parkinsonism. When Parkinsonism is precipitated by a different etiology, it is termed secondary Parkinsonism. Tumors are a rare cause of secondary Parkinsonism, meningiomas, gliomas, and cavernomas being the most common [1-6]. Tumors in different cortical and subcortical structures have been reported to cause secondary Parkinsonism [6].

Craniopharyngioma is a rare central nervous system (CNS) tumor with a prevalence of 0.5 to 2 cases per million persons per year. It usually arises in the suprasellar region and can extend to involve multiple brain segments, producing a variety of symptoms. The most common manifestations of craniopharyngioma are elevated intracranial pressure, visual impairments, and endocrine deficiencies [2]. Secondary Parkinsonism due to craniopharyngiomas is rare, and only a single example was reported by Parvaresh in 2015 [7]. In that case, Parkinsonism was reversible, and levodopa was discontinued after one week. We describe a unique case of a patient developing irreversible levodopa-responsive Parkinsonism following resection of craniopharyngioma. This case has not been presented at a national or international conference.

## Case presentation

A 42-year-old woman presented to the Emergency Department with a two-month history of progressive resting tremors in her hands. 

Her medical history was relevant for cutaneous lupus erythematosus and a papillary craniopharyngioma resected four months prior to the presentation. The postoperative course was complicated by diabetes insipidus, panhypopituitarism, and severe delirium, which were managed with DDVAP, levothyroxine, haloperidol, and aripiprazole. Her original preoperative presentation was significant for two weeks of fatigue, bilateral blurry vision, bilateral lower extremity weakness, and urinary incontinence. Family history was noncontributory.

The neurological examination showed hypophonic speech and an intermittent resting tremor. The tremor was more noticeable in her left upper extremity. Provoked mental math induced tremor spread to the head and lower extremities. There was cogwheel rigidity with more prominence in the right upper extremity compared to the left. She also exhibited bradykinesia on hand grips, finger taps, and toe taps (Video 1).

**Video 1:** Neurological exam on presentation. Neurological exam on presentation shows hypophonic speech, intermittent resting tremor, cogwheel rigidity, and bradykinesia on hand grips, finger taps, and toe taps (not pictured).

**Figure 1:**
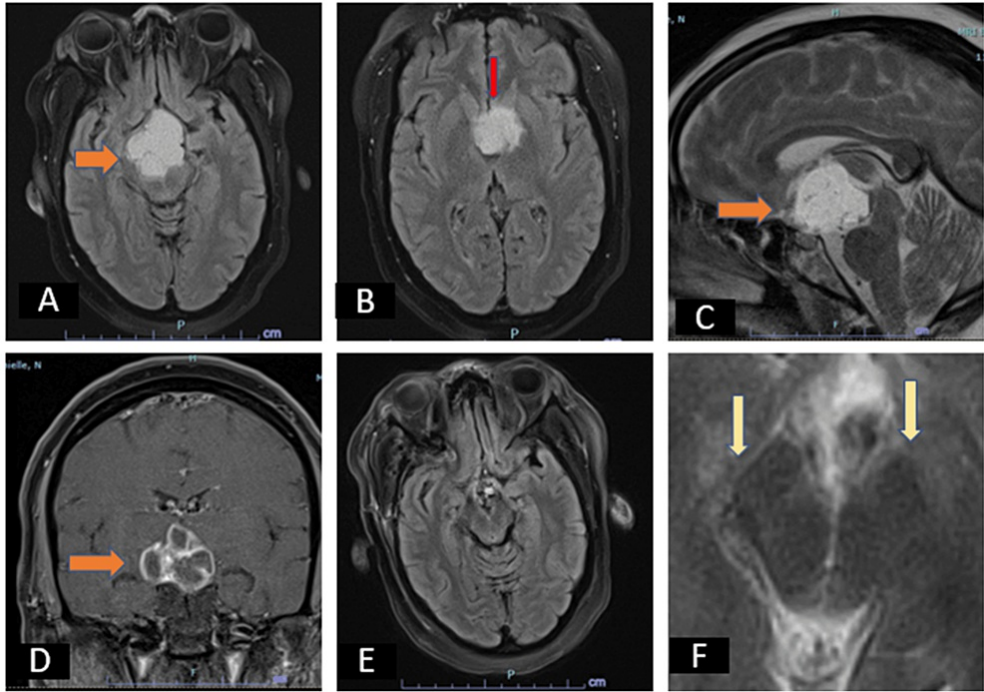
Pre and postoperative brain MRI (T2/SWI) A: T2 Flair axial sequence of preoperative brain MRI showing a craniopharyngioma compressing the midbrain. B: T2 Flair axial sequence of preoperative brain MRI showing a craniopharyngioma compressing the caudates. C: T2 Sagittal T2 postcontrast sequence of preoperative brain MRI showing a craniopharyngioma compressing the midbrain. D: T1 Coronal postcontrast sequence of preoperative brain MRI showing a craniopharyngioma compressing the midbrain. E-F: Postoperative Brain MRI showing loss of normal swallow tail in T2/SWI (F, yellow arrows), with expected surgical changes in brain MRI and no further compression in midbrain (E) MRI: Magnetic resonance imaging; T2/SWI: T2/susceptibility-weighted imaging

The initial diagnosis was drug-induced Parkinsonism given that the patient had been taking haloperidol 10 mg and aripiprazole 10 mg for about four months for delirium. She had originally been recommended to take those medications as needed, but further questioning revealed that she was taking them daily. Knowing that she had a craniopharyngioma measuring 3.8 x 2.3 x 2.0 cm with midbrain compression on preoperative MRI, we decided to obtain a brain MRI to assess the integrity of the anterior midbrain and nigrostriatal system. The new MRI was significant for the loss of normal swallow tail in T2/SWI with expected surgical changes (Figure 1).

Haloperidol and aripiprazole were stopped, and benztropine 1 mg BID was started without improvement in Parkinsonian features. Given the compressive features of patient’s craniopharyngioma, we decided to start carbidopa/levodopa 25/100 mg TID. We saw moderate improvement of bradykinesia, resting tremors, and gait instability.

**Figure 2:**
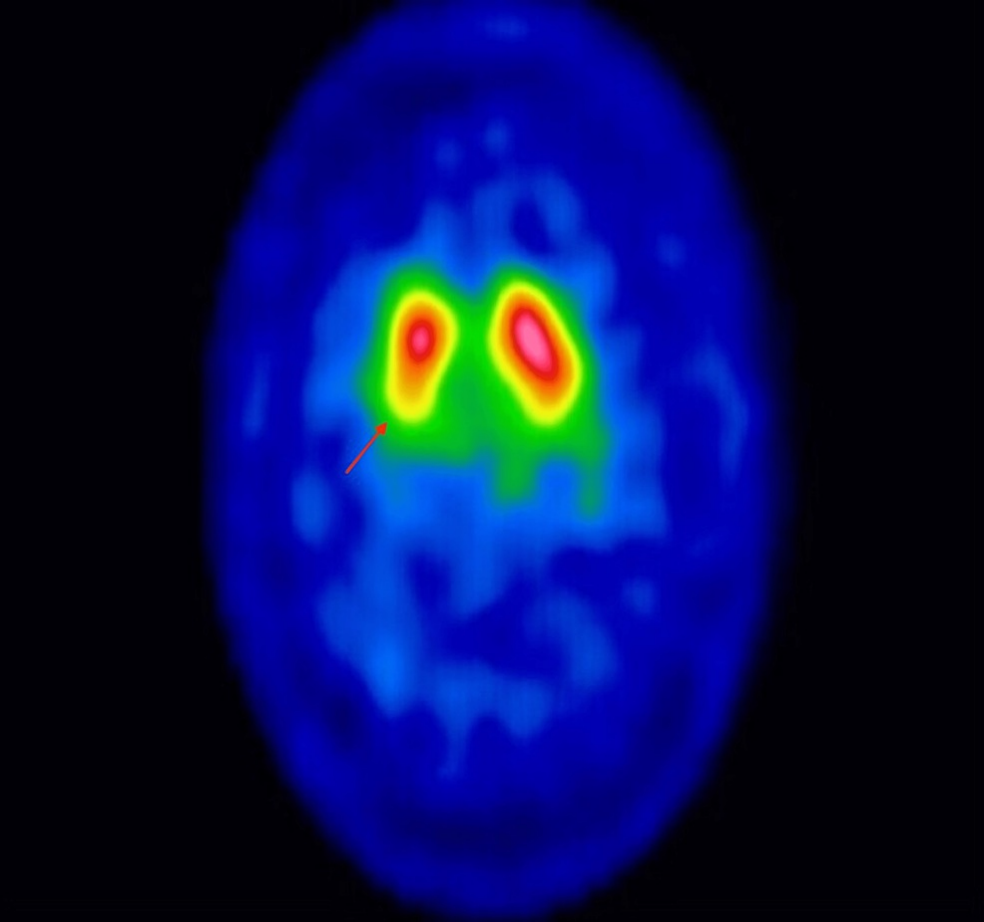
DaT Scan DaT Scan shows an asymmetric decrease in uptake in the striatum that is more predominant in the right caudate and putamen (indicated by the red arrow) compared with the left. DaT: Dopamine transporter

In this case, the use of antipsychotics represented a challenge as it could have been masking an underlying Parkinsonian syndrome caused by the brain tumor. To confirm the diagnosis of neurodegenerative parkinsonism, we obtained a dopamine transporter (DaT) scan on an outpatient basis after starting treatment with carbidopa/levodopa. It demonstrated an asymmetric bilateral decrease uptake of radiotracer more predominant in the right caudate and putamen (Figure 2), which confirmed our suspicion of nigrostriatal dysfunction and ruled out drug-induced Parkinsonism in which the DaT scan is normal. The patient was followed up in the clinic at five and eight months, and carbidopa/levodopa was titrated up with an excellent clinical response (Video 2).

**Video 2:** Neurological exam eight months after starting treatment. Neurological exam eight months after starting treatment shows improvement in bradykinesia and tremors.

.

## Discussion

Craniopharyngioma is a rare primary CNS tumor. It can extend to multiple brain structures, with most symptoms due to the mass effect [2]. Secondary Parkinsonism is a rare manifestation of brain tumors, more commonly in gliomas and meningiomas. Per the literature, the predominant mechanism is compression of the midbrain or basal ganglia [3]. 

Secondary Parkinsonism due to craniopharyngiomas is rare, and only a single case was reported by Parvaresh in 2015 [7]. In that case, the patient had hydrocephalus, and Parkinsonism resolved after surgical intervention (shunt placement), and dopamine replacement therapy was discontinued within one week [6]. In comparison, our patient did not have hydrocephalus and she developed irreversible symptoms likely due to long-term mass effect over the anterior midbrain and caudate, requiring maintenance dopamine-replacement therapy. Another etiology could be ischemia as there is literature supporting that patients with craniopharyngiomas develop severe morphological changes in the tunica intima, especially in the basal arteries. These changes could have led to a progressive deficit in the blood supply and caused hypoperfusion of the midbrain in the postoperative period. Notably, purely vascular etiologies of Parkinsonism have normal DaT scans [8].

In this case, the craniopharyngioma extended to compress substantia nigra and striatum bilaterally, precipitating irreversible secondary Parkinsonism. A DaT scan was used to confirm nigrostriatal dopaminergic neurodegeneration, which is seen in Parkinsonian syndromes [1,7]. Although complications of craniopharyngioma have been described in the literature, no similar case of irreversible secondary Parkinsonism supported by the DaT scan has been published, which is the main strength of our case report. The first limitation of this case is the lack of additional published examples of brain tumor-induced Parkinsonism with nigrostriatal damage supported by the DaT scan. This limits our ability to determine the time required to develop enough damage to be noticeable on the DaT scan. The second limitation is that vessel imaging was not done during admission, which limits our ability to rule out postoperative vascular changes as a contributor to this presentation. However, this is less likely given the degree of irreversible compression from the tumor.

## Conclusions

This case report demonstrates a rare case of papillary craniopharyngioma causing secondary Parkinsonism due to likely compression on the midbrain and substantia nigra. Diagnosis and management of such cases can be challenging, especially if there are overlapping factors like the use of antipsychotics. DaT scan is used to confirm the loss of neurons in the substantia nigra and to differentiate from purely vascular causes of Parkinsonism, where dopamine uptake remains normal. We hope to encourage the early consideration of brain tumors as a cause of Parkinsonism in young patients as an early surgical intervention might be curative.
